# Counterclockwise Drilling with Different Tapered Drills Condenses the Implant Bed—An Optical Coherence Tomography In Vitro Study

**DOI:** 10.3390/medicina57090940

**Published:** 2021-09-06

**Authors:** Rafael Delgado-Ruiz, Mina Mahdian, Ilyasse Benezha, Georgios Romanos

**Affiliations:** 1 Department of Prosthodontics and Digital Technology, School of Dental Medicine, Stony Brook University, New York, NY 11794-8712, USA; mina.mahdian@stonybrookmedicine.edu (M.M.); ilyassebenezha@gmail.com (I.B.); 2 Department of Periodontology, School of Dental Medicine, Stonybrook University, New York, NY 11794-8712, USA; georgios.romanos@stonybrookmedicine.edu

**Keywords:** implant bed preparation, bone condensation, counterclockwise drilling, tapered drills, optical coherence tomography (OCT)

## Abstract

*Background and Objectives:* To evaluate the condensation and the microarchitecture of implant bed walls of sites prepared with counterclockwise drilling with tapered implant drills using optical coherence tomography. *Materials and Methods*: Four drill designs with different wall and tip angles were used. Polyurethane laminas resembling type IV bone microarchitecture were superimposed and clamped with a vice to simulate the coronal, middle, and apical aspects of the implant site. Twenty implant beds were prepared at 1200 rpm in clockwise (control) and counterclockwise (test) directions (N = 160). Optical coherence tomography (OCT) was used to evaluate the condensation and microarchitecture characteristics of the implant bed walls. The relative condensation was calculated using the Image J software Bone application. The microarchitecture was evaluated in reconstructed 3D volumes in XY, XZ, and YZ sections. Statistical analysis was performed using one-way ANOVA. Dunnet test was applied to determine differences between groups. Significance was set as *p* < 0.05. *Results*: Counterclockwise drilling (Test) condensed and changed the microarchitecture of the apical regions for all the implant beds in all of the groups when compared to clockwise drilling (control). The apical region of test groups showed the highest relative bone condensation (*p* = 0.026) when compared to controls. *Conclusions*: The direction of rotation (counterclockwise drilling) and not the design of tapered drills (tip and wall angles) is responsible for the condensation at the apical area observed in polyurethane blocks. The OCT method can be used for the evaluation of changes in density and microstructure of polyurethane blocks.

## 1. Introduction

The evaluation of bone quantity (BQT) and bone quality (BQL) is a fundamental step during implant planning. The BQT analysis evaluates the bone availability for the placement of dental implants outlined by different anatomical structures [1]. The BQL is a relatively vague term that assesses the characteristics of the cortical bone (thickness, density, and porosity) and cancellous bone (trabecular number, thickness, and connectivity) [2].

These factors can predict dental implant stability and serve as relative prognosis factors in cases of immediate implant loading [3]. Regarding BQL, it appears that implant survival is similar in patients with low and average bone density [4]. However, late implant failures have been correlated to poor quality of cancellous bone [5], patients with osteoporosis have shown increased peri-implant bone loss compared with healthy patients after 5 years [6], initial low BQL has been linked with increased bone remodeling around dental implants [7], and implants inserted in bone of poor quality present an increased risk of failure compared to implants inserted in bone of good quality [8].

Furthermore, when dental implants are inserted in low-density bone (type IV bone—Lekholm and Zarb classification), characterized by large trabecular spaces and thin cortical bone [9], the risk of implant micromovement increases [10], and micromotion above 150 microns can result in the formation of a fibrous tissue that hinders the implant’s osseointegration [11,12].

Methods to improve the BQL to reduce implant micromovement include reducing the trabecular spacing, increasing the bone compaction/condensation of the implant bed walls, using implant designs with increased engagement with the adjacent bone (tapered implants and progressive/aggressive thread designs), and increasing the implant length and diameter [13].

Conservative implant bed preparation techniques with minimal bone removal or no-bone removal (instead of cutting bone, displacing bone laterally and apically) can be applied to improve poor BQL [14,15,16]. These techniques include osteotomes [17,18], bone compactors [19,20,21], and the osseodensification technique [22,23,24]. Bone condensation and compaction are achieved, provided the bone tissue’s viscoelastic properties that allow some degree of bone deformation (due to the collagen content), bone compaction, and some degree of bone fractures, which result in the increased bone density of the walls of the treated area [17,18,19,20]. The osseodensification technique also displaces and condenses the bone laterally and apically; the displaced bone particles can act as an autograft; and, primary, implant stability can be increased [22,23,24].

It is unclear if the drill bit design (walls angle and tips angle) or the direction of drill rotation is responsible for the bone structure changes and the bone condensing observed with osseodensification techniques. Moreover, in theory, it could be hypothesized that any tapered drill could modify the BQL (bone densification, produced by thicker/denser bed walls and thicker/denser apical bone of the implant bed, and potential changes in the trabecular spacing) when drilling in a counterclockwise direction.

This study aimed to analyze the effect of clockwise versus counterclockwise drilling with tapered drills with different designs on the implant bed wall density and trabecular structure of implant beds prepared in polyurethane blocks representing type IV bone density using OCT methods.

## 2. Materials and Methods

**Figure 1:**
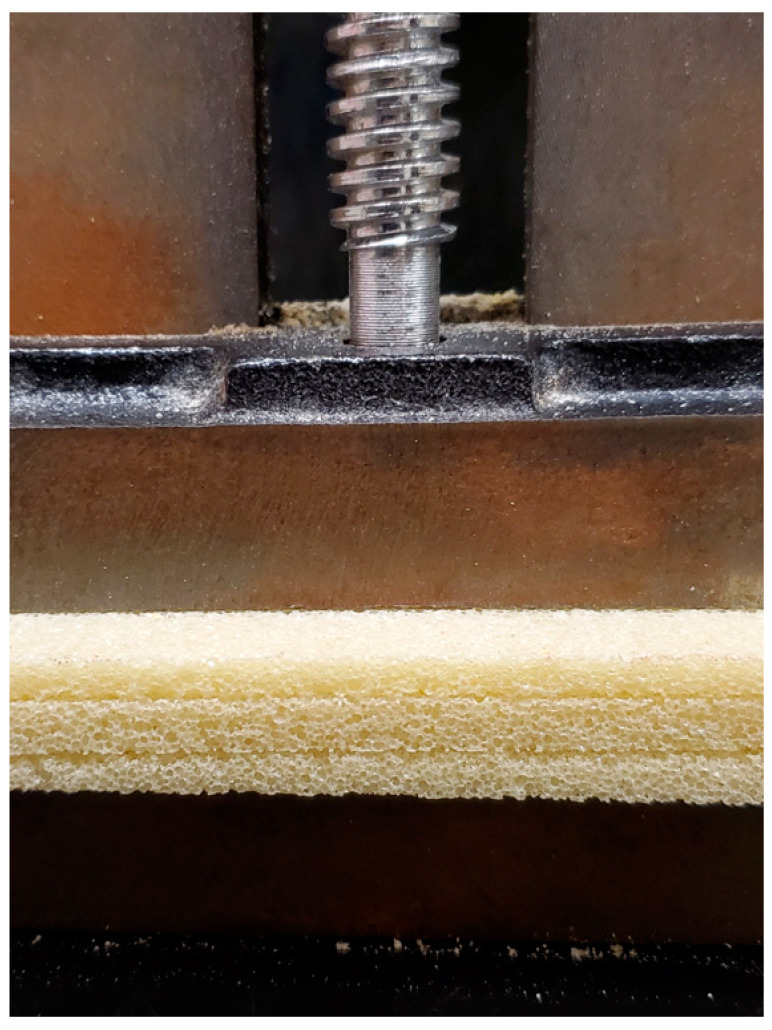
Polyurethane foam laminas. This image illustrates the setting of three superimposed laminas clamped with a vice. The thickness of each lamina is 3.3 mm, and the total thickness of the three laminas all together was ±10 mm.

Synthetic bone laminas resembling type IV bone (Sawbones, Pacific Research Laboratories, Vashon, CA, USA) made in polyurethane foam with a spaced trabeculae structure and a density of 33 pounds per cubic foot (PCF) (0.38 g/cm^3^) were used. Three laminas were superimposed and clamped with a vice to simulate the coronal, middle, and apical aspects of the future implant site (Figure 1).

**Figure 2:**
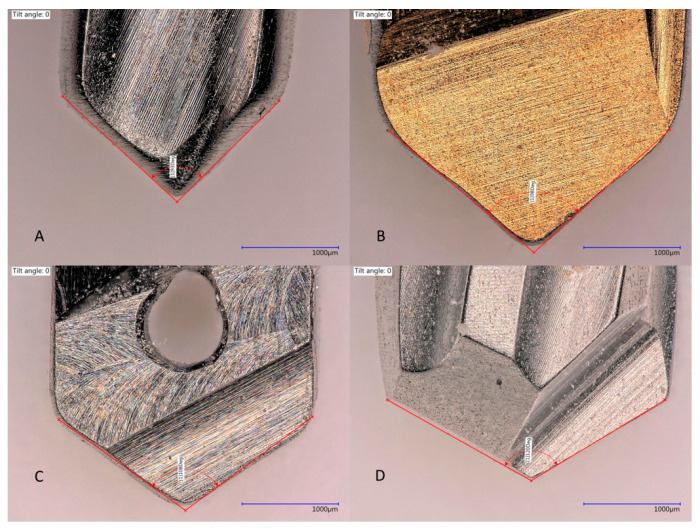
Drill tip angles. Composition image of the four drill tips used in this experiment. (**A**) Anker tapered drill tip, angle: 93 degrees; (**B**) Megagen tapered drill tip, angle: 98 degrees; (**C**) Nobel Biocare tapered drill tip, angle: 108 degrees; and (**D**) Densah tapered drill tip, angle: 120 degrees.

**Figure 3:**
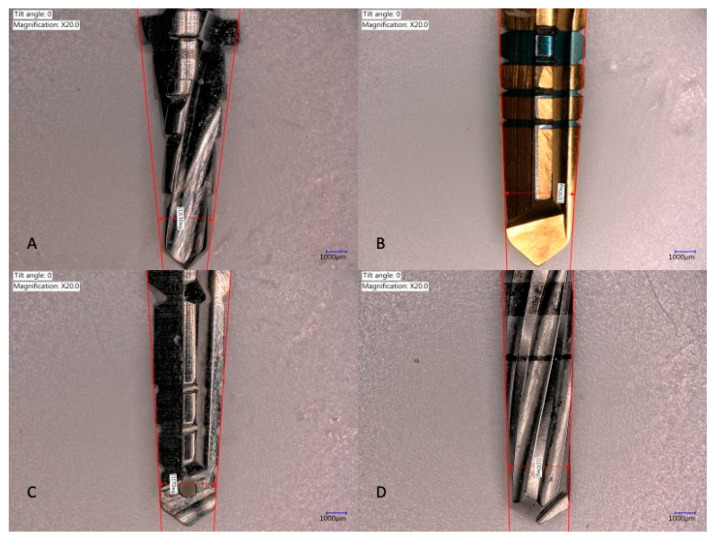
Drill wall angles. Composition image of the four drills used in this experiment. (**A**) Anker tapered drill walls, angle: 13 degrees; (**B**) Megagen tapered drill walls, angle: 5 degrees; (**C**) Nobel Biocare Tapered drill walls, angle: 7 degrees; and (**D**) Densah tapered drill walls, angle: 3 degrees.

The total thickness of three coupled laminas was ±10 mm. Tapered implant drills from four implant systems were used: Anyone^®^ tapered drills (Megagen, Seoul, Korea), Replace Select^®^ tapered drills (Nobel Biocare, Yorba Linda, CA, USA), Anker SB II^®^ drills (Alliance Global Technology, Kaohsiung, Taiwan), and Densah^®^ drills (Versah, Jackson, MI, USA). The diameter and length of the drills were comparable, but the wall, tip angles, and design were different (Table 1, Figure 2 and Figure 3).

### 2.1. Drilling Parameters

The following parameters were standardized: drilling speed 1200 rpm, drilling depth of ±10 mm, and profuse irrigation. In total, 160 implant bed preparations were completed, with 40 implant bed preparations per drill group (20 clockwise and 20 counterclockwise).

#### 2.1.1. Clockwise Drilling Method (Control)

The drill rotation was set in the clockwise direction. A pumping method was used to apply slight downward pressure to produce perforation of the bone laminas followed by drill withdrawal to allow stress relief until the drill reached a depth of ±10 mm.

#### 2.1.2. Counterclockwise Drilling Method (Test)

The drill rotation was set in the counterclockwise direction. A slight downward pressure was applied to allow the perforation of the bone laminas; then, the drills gradually advanced into the implant bed preparation. Afterwards the drills were pulled out (for stress relief), and then downward pressure was repeatedly applied (in/out fashion) until a drilling depth of ±10 mm was achieved.

### 2.2. Evaluation of the Bone Density and Bone Structure

After the completion of the implant bed preparations, the bone laminas were separated, and the relative bone density and bone structure characteristics were analyzed in each lamina for each drill group. The areas of analysis were the 3 mm coronal laminas, 3 mm middle laminas, and 3 mm apical laminas that simulated the implant bed.

The OCTG-900 spectral-domain optical coherence tomography (OCT) device (Thorlabs, Newton, NJ, USA) was used for the evaluation of the bone density and structure. Cross-sectional (2D) and volumetric (3D) data of the implant beds were obtained using an A-scan depth of 2.9 mm and a pixel size of 21.04 ± 2.32 µm to achieve a transverse resolution of 4 μm and a vertical resolution of 6 μm. The refractive index of the polyurethane foam was set as 1.5, and the scanning was completed at low-speed and high-sensitivity recording (5.5 kHz). The light intensity limits were a lower value (LV) of 22.0 and a higher value (HV) of 80.3.

For the cross-sectional scanning, a 10 mm length scanning line was traced. The line passed through the center of the implant bed preparation, dividing the implant bed into two equal semicircles. The line was extended by 3 mm per side (thus extending the scanning line by 3 mm to both sides of the implant beds). Line scanning (for the cross-sectional evaluations) was completed for each osteotomy (160 times in total).

For 3D volumetric scanning, squared areas of 10 mm × 10 mm contained in the center of each of the implant bed preparations were traced. In total, 160 volumetric analyses were completed. A-scan/line rates were set as 5–248 kHz with a 101–102 dB sensitivity for cross-sectional and volumetric evaluations.

**Figure 4:**
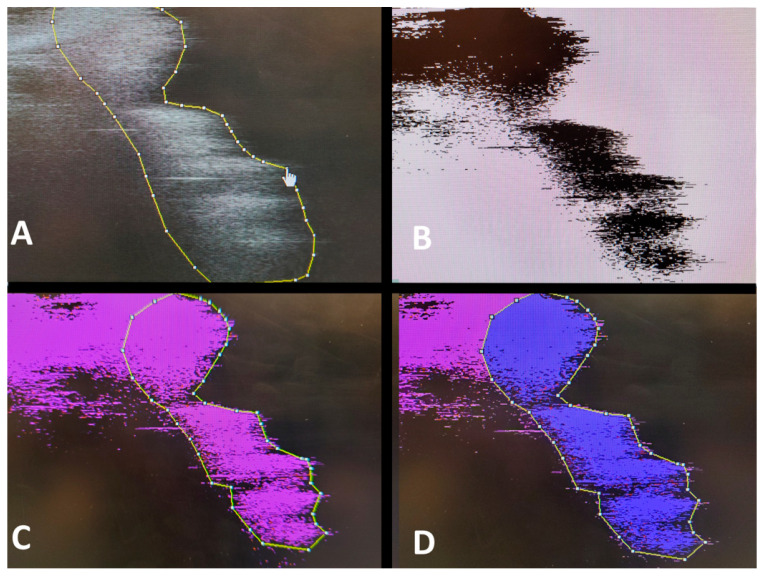
Image of processing for measuring. (**A**) Implant bed wall region of interest marked with a polygon. (**B**) Transformation in an 8-bit image. (**C**) Thresholding of the region of the interest based on the pixel color. (**D**) Region of interest structured and measured.

The following quantitative and qualitative variables were evaluated:

- Implant bed wall condensation/density (quantitative): Two cross-sections passing through the center of the implant bed preparation were obtained, thus allowing the evaluation of the four walls of the implant bed preparation. Afterward, the recorded files were transferred to the analysis software Image J (National Institutes of Health, Bethesda, MD, USA). Pixel size was determined based on the refractive index of the trabecular bone and the trabecular spacing. The implant bed wall with high density was characterized by high refraction, and a region of interest (ROI) was traced per section. The percentage of highly refractive bone was traced, and the percentage of bone in relation to the whole area was extracted (Figure 4A–D). Qualitative changes, including trabecular space narrowing and trabecula fracture, as well as no change, were described. The mean condensation of each osteotomy at the coronal, middle, and apical region were collated per group, and mean ± SD deviations were recorded for each drill design. Results are expressed in percentages.

**Figure 5:**
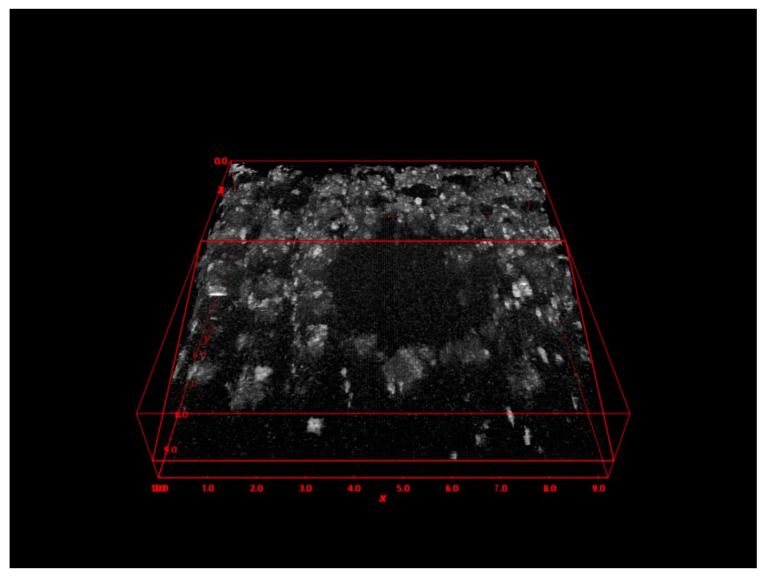
Volume reconstruction for the qualitative evaluation of the microarchitecture. Scanning was completed in a volume of 10 mm × 10 mm. This is a 3D image of the coronal section of the Densah burs group in counterclockwise drilling.

Trabecular structural changes (qualitative): The trabecular structure adjacent to the 3 mm of the perimeter of the implant bed preparation was evaluated. ThorImageOCT software (Thorlabs, Newton, NJ, USA) was used to mark a square containing the implant bed preparation. The dimensions of the square were 10 mm by 10 mm. The trabecular structure characteristics surrounding 360 grades of the implant bed site were detected. Vertical and horizontal scanning of the area was completed to create a 3D reconstruction (Figure 5). Thus, 3D evaluation of the implant bed walls and proper description of the trabecular structure characteristics were completed.

### 2.3. Statistical Analysis

The sample size of a confidence level of 95%, with an odds ratio of 10% and a power of 90%, was established as *n* = 20. Twenty implant bed preparations in clockwise rotation and twenty preparations in counterclockwise rotation for each implant drill geometry were prepared. Forty osteotomies were completed per group for a total sample size of N = 160 osteotomies. The normality of the samples was evaluated with the Shapiro–Wilk test, and one-way ANOVA with Dunnet post-test were used to compare differences between groups. The significance of differences was set as *p* < 0.05.

## 3. Results

### 3.1. Coronal

**Figure 6:**
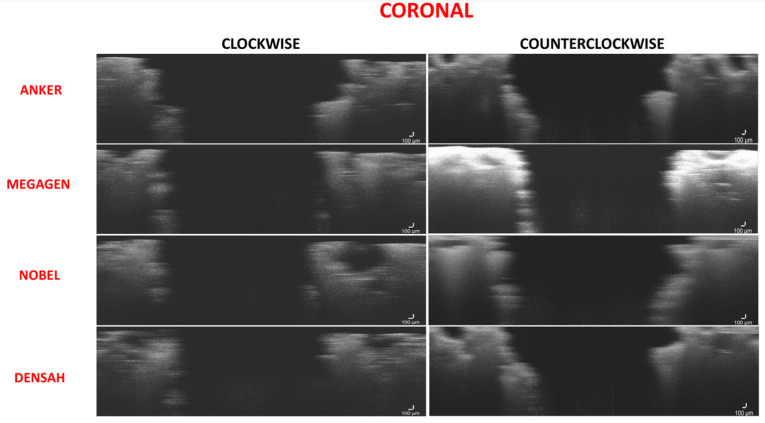
Figure composition showing representative samples of 2D sections at the coronal region for clockwise and counterclockwise drilling for all of the drill designs. Clockwise and counterclockwise drilling produced microfractures of the implant bed walls. Some localized thickening was observed in both drilling directions. However, wall density was not increased in any of the drilling directions.

**Figure 7:**
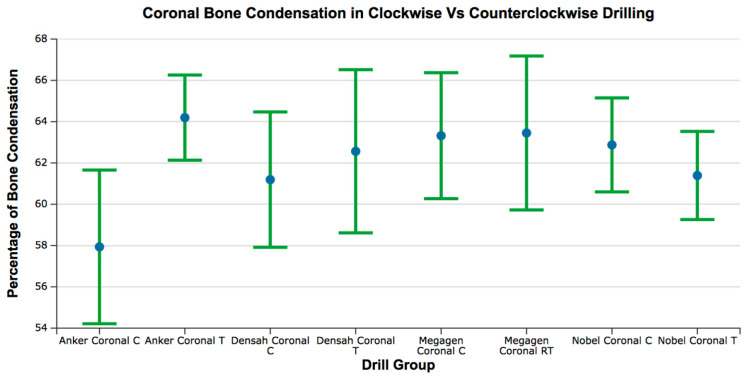
Box plot for the percentage of bone condensation at the coronal area for all groups. C = clockwise drilling; T = counterclockwise drilling. The Anker drill group showed a lower mean value; however, the statistical analysis did not show significant differences.

The range of bone condensation at the coronal section produced by tapered drills with clockwise rotation (61.19 ± 4.58%) was similar to that produced by tapered drills with counterclockwise rotation (62.57 ± 5.52%). When comparing all of the tapered drills and drilling directions at the coronal area, only the Anker drill showed significant increased bone condensation with counterclockwise drilling when compared to clockwise drilling (*p* = 0.04) (Figure 6 and Figure 7; Table 2 and Table 3).

### 3.2. Middle

**Figure 8:**
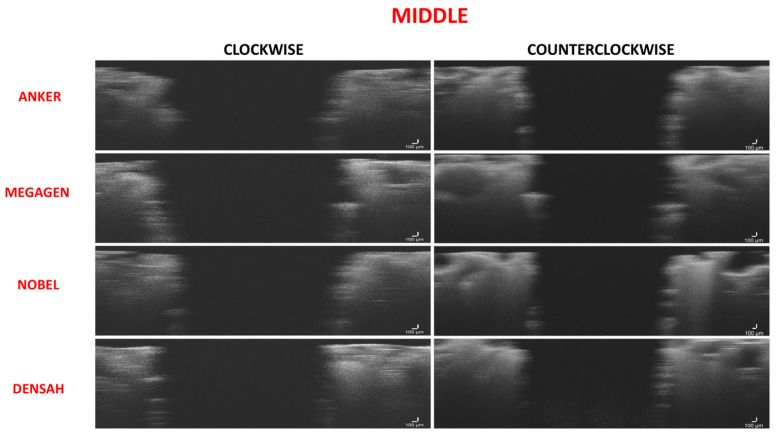
Figure composition showing representative samples of 2D sections at the middle region for clockwise and counterclockwise drilling for all of the drill designs. Microfractures were observed in all the implant bed walls. Some of the empty spaces of the cancellous area were exposed to the implant bed intaglio. Wall density appeared the same for both drilling directions.

**Figure 9:**
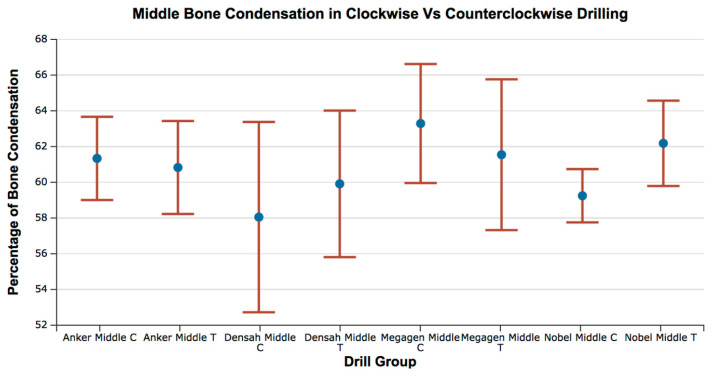
Box plot for the percentage of bone condensation at the middle area for all groups. C = clockwise drilling; T = counterclockwise drilling.

The range of bone condensation at the middle section produced by tapered drills with clockwise rotation (58.05 ± 7.45%) was similar to that produced by tapered drills with counterclockwise rotation (59.91 ± 5.73%) (Table 4). When comparing all of the tapered drills and drilling directions at the coronal area, there was no difference in the percentage of bone condensation between counterclockwise drilling and clockwise drilling (*p* > 0.05) (Figure 8 and Figure 9; Table 4 and Table 5).

### 3.3. Apical

**Figure 10:**
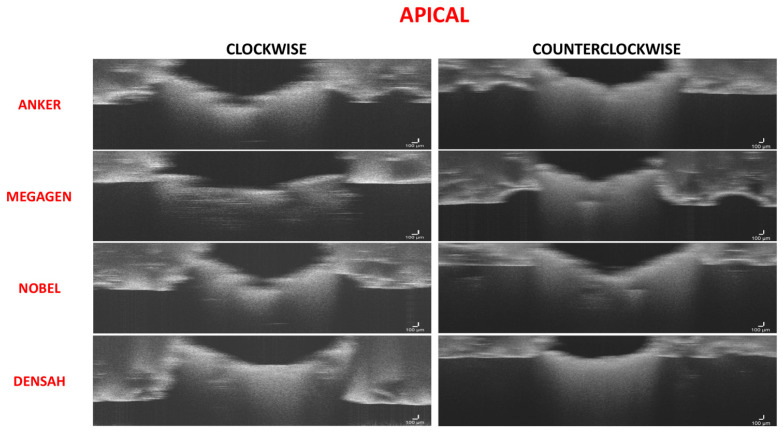
Figure composition showing representative samples of 2D sections at the apical region. Both groups showed slightly increased density at the apical area in clockwise rotation. The counterclockwise drilling showed the highest bone density for all of the drill designs.

**Figure 11:**
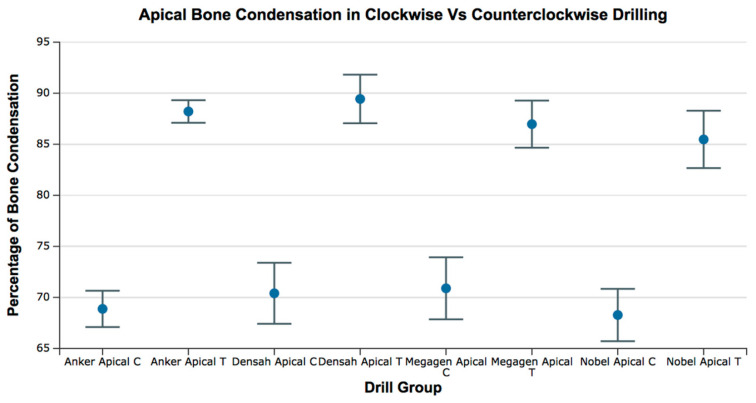
Box plot for the percentage of bone condensation at the apical area for all groups. C = clockwise drilling; T = counterclockwise drilling.

The range of bone condensation at the apical section produced by tapered drills with counterclockwise rotation (87.52 ± 3.01%) was higher than that with tapered drills with clockwise rotation (69.61 ± 3.625%). When comparing all of the tapered drills and drilling directions at the apical area, there was a significant difference in the percentage of bone condensation between counterclockwise drilling and clockwise drilling (*p* < 0.05) without differences between drill designs (*p* > 0.05) (Figure 10 and Figure 11; Table 6 and Table 7).

Clockwise drilling with tapered drills with various tip and wall angles did not produce condensation of the implant bed walls, nor did it change the trabecular spacing. Meanwhile, counterclockwise drilling with tapered drills produced condensation of the implant bed at the apical area and produced a reduction in the trabecular spacing. The degree of condensation at the apical area of implant beds prepared with counterclockwise drilling was similar for all of the drill designs and was not related with variations in tip and wall angles.

## 4. Discussion

The goal of the present study was to determine any possible differences between tapered drills with different designs (tips and wall angles) and different directions of rotation (clockwise versus counterclockwise) and to evaluate the changes of the density of implant beds prepared in polyurethane blocks representing type IV bone.

The results of the present work showed that the direction of rotation, but not the drill design (tips and wall angles), increased the density of the implant bed. The bone density was significantly increased at the apical area in all the groups with counterclockwise drilling rotation, while no changes in bone wall density were observed at the coronal and middle regions.

The influence of the direction of rotation of implant drills on the bone density was first described by Huwais and Meyer [22]. The authors used a porcine tibia model and a tapered drill with multiple flutes that rotated clockwise or counterclockwise. The authors noted that counterclockwise drilling produced increased bone density around the implant osteotomies evaluated with microcomputed tomography (µCT), among other findings. The authors identified compacted bone with a thickness of 0.1 mm to 0.3 mm at the edges of the osteotomy and 0.5 mm to 1.0 mm thickness at the apical area of the osteotomy.

However, the authors did not compare their drill design with other drill designs. Furthermore, Antonelli et al. [21] and Delgado-Ruiz et al. [25], showed in experimental models in pig ribs that the counterclockwise drilling technique increased the bone density of the implant bed walls and that bone density was higher at the apical region (evaluated with µCT). Their results are in agreement with the results of the present work in regard to the apical area; however, no evidence of bone condensation was observed with any of the drills and rotation direction used in the present work. These differences could be produced by the different viscoelastic properties of pig bone when compared to the viscoelastic properties of polyurethane blocks [26].

During counterclockwise drilling, fracture and a simultaneous displacement of the bone occurred in the apical direction. Meanwhile, counterclockwise drilling produced a pure fracture phenomenon of the implant bed walls without lateral displacement.

This difference explains the increased bone condensation observed at the apical region but not at the bed walls, which is in agreement with the theories of Frost [27,28].

Currently, different techniques are used to evaluate bone quantity and quality, including dental radiographs [29,30], cone-beam computed tomography (CBCT) [29], and microcomputed tomography (micro-CT) [30]. Recently, non-ionizing methods, such as magnetic resonance imaging (MRI) [31,32] and optical coherence tomography (OCT) [33], were introduced as potential methods for the evaluation of bone characteristics.

An OCT device was used in the present study to evaluate the structure and condensation of the trabecular structure of polyurethane blocks. This method was selected given its high resolution, high contrast, and the three-dimensional reconstruction capabilities that OCT systems have shown [33,34]. Moreover, this method allowed for the preparation of cross-sectional images to disclose the internal structure of the polyurethane blocks non-destructively [35]. Furthermore, bone mineral density has been evaluated by light scattering methods (which are the basis of OCT), and it was found that OCT is efficient in evaluating and detecting regional changes of bone density [36].

OCT is comparable with the micro-CT technique [37], with differences in higher penetration depth for micro-CT (the OCT device penetration depth is limited to between 2.5 mm to 3 mm) and better contrast and resolution for OCT [37]. To overcome the depth penetration limitations of OCT in the present study, bone laminas with a width of 3 mm were used. Thus, the evaluations were always within the range of a resolution depth of 3 mm of the OCT. In addition, polyurethane blocks with semi-transparent qualities and a known refractive index were used.

One drawback of the present work is that the trabecular pattern of the polyurethane foams is too homogeneous. As a result, it does not reproduce the heterogeneous structure of the human trabecular bone or other experimental animal models (pig and bovine ribs). This issue could be minimized by using layered or multi-structured (cortico-cancellous) polyurethane foams in future studies.

To date, experimental studies have been focused on the comparison of counterclockwise drilling with a single type of drill design (multifluted tapered drills). The present study results demonstrated that the tapered drill design has no influence on bone condensation. Instead, the direction of the rotation (counterclockwise drilling) of tapered drills with various tips and wall angles is responsible for the observed apical bone condensation.

## 5. Conclusions

Within the limitations of this experimental in vitro study, it can be concluded that the direction of rotation (counterclockwise drilling) and not the design of tapered drills (tip and wall angles) is responsible for the condensation at the apical area observed in polyurethane blocks. The OCT method can be used for the evaluation of changes in the density and structure of polyurethane blocks.

## Figures and Tables

**Table 1:** Drill manufacturer, drill type, reference, wall angle, and tip angle.

Manufacturer	Drill	Reference	Wall Angle	Tip Angle
Anker	SB II	SBS 4010	13	93
Megagen	Anyone	SD4218S	5	98
Nobel Biocare	Replace Select	29371	7	108
Densah	Versah	VS3238	3	120

**Table 2:** Descriptive statistics. Relative bone condensation at the coronal region.

Group	Mean	Minimum	Maximum
Densah Coronal C	61.19	55.15	68.03
Densah Coronal T	62.57	50.12	69.41
Nobel Coronal C	62.87	56.19	66.87
Nobel Coronal T	61.39	57.61	66.28
Megagen Coronal C	63.32	57.25	70.58
Megagen Coronal T	63.45	55.89	71.07
Anker Coronal C	57.94	46.10	64.58
Anker Coronal T	64.20	59.13	69.71

**Table 3:** Statistical comparison of the relative bone condensation at the coronal region for all of the groups. No significant differences were observed between groups. T = Test, C = Control, SE = Standard Error, t = test, *p* = *p* value.

Dunnett Post Hoc Comparisons—CORONAL				
	Mean Difference	SE	t	*p*
Anker Coronal T—Anker Coronal C	6.260	1.945	3.219	0.011
Densah Coronal C—Anker Coronal C	3.258	1.945	1.675	0.394
Densah Coronal T—Anker Coronal C	4.632	1.945	2.382	0.101
Megagen Coronal C—Anker Coronal C	5.386	1.945	2.769	0.040
Megagen Coronal T—Anker Coronal C	5.518	1.945	2.838	0.033
Nobel Coronal C—Anker Coronal C	4.940	1.945	2.540	0.070
Nobel Coronal T—Anker Coronal C	3.459	1.945	1.779	0.333

**Table 4:** Descriptive statistics. Relative bone condensation at the middle region.

Group	Mean	Minimum	Maximum
Densah Middle C	58.05	48.15	68.90
Densah Middle T	59.91	48.34	64.68
Megagen Middle C	63.29	54.23	67.95
Megagen Middle l T	61.54	52.21	74.41
Nobel Middle C	59.25	54.58	61.51
Nobel Middle T	62.18	56.54	66.88
Anker Middle C	61.34	54.95	66.11
Anker Middle T	60.83	54.06	66.42

**Table 5:** Statistical comparison of the relative bone condensation at the middle region for all groups. No significant differences were observed between groups. T = Test, C = Control, SE = Standard Error, *t* = test, *p* = *p* value.

Dunnett Post Hoc Comparisons—MIDDLE				
	Mean Difference	SE	t	*p*
Anker Middle T—Anker Middle C	−0.509	2.145	−0.237	1.000
Densah Middle C—Anker Middle C	−3.286	2.145	−1.532	0.488
Densah Middle T—Anker Middle C	−1.425	2.145	−0.664	0.979
Megagen Middle C—Anker Middle C	1.952	2.145	0.910	0.902
Megagen Middle T—Anker Middle C	0.206	2.145	0.096	1.000
Nobel Middle C—Anker Middle C	−2.090	2.145	−0.974	0.870
Nobel Middle T—Anker Middle C	0.844	2.145	0.393	0.999

**Table 6:** Descriptive statistics. Percentage of relative bone condensation at the apical region.

Group	Mean	Minimum	Maximum
Densah Apical C	68.88	64.98	72.09
Densah Apical T	88.21	85.86	90.81
Megagen Apical C	70.89	65.13	76.43
Megagen Apical T	86.97	79.88	91.03
Nobel Apical C	70.40	62.03	74.99
Nobel Apical T	89.44	85.64	96.05
Anker Apical C	68.27	63.4	73.87
Anker Apical T	85.47	76.43	89.87

**Table 7:** Statistical comparison of the relative bone condensation at the apical region for all groups. The test groups (counterclockwise drilling) showed increased relative bone condensation compared to the controls (clockwise drilling). T =Test, C = Control, SE = Standard Error, t = test, *p* = *p* value.

Dunnett Post Hoc Comparisons—APICAL				
	Mean Difference	SE	t	*p*
Anker Apical T—Anker Apical C	19.335	1.531	12.626	<0.001
Densah Apical C—Anker Apical C	1.527	1.531	0.997	0.858
Densah Apical T—Anker Apical C	20.560	1.531	13.426	<0.001
Megagen Apical C—Anker Apical C	2.015	1.531	1.316	0.644
Megagen Apical T—Anker Apical C	18.092	1.531	11.814	<0.001
Nobel Apical C—Anker Apical C	−0.601	1.531	−0.393	0.999
Nobel Apical T—Anker Apical C	16.597	1.531	10.838	<0.001

## Data Availability

Data will be made available upon request.

## Abbreviations

ANOVA	Analysis of variance
BQL	Bone quality
BQT	Bone quantity
g/cm^3^	Grams/cubic centimeter
μm	Micrometers
kHz	Kilohertz
OCT	Optical coherence tomography
PCF	Pound per cubic foot
Rpm	Revolutions per minute

## References

[1] Jaffin R., Berman C. (1991). The excessive loss of Branemark fixtures in type IV bone: A 5-year analysis. J. Periodontol..

[2] Ribeiro-Rotta R., Lindh C., Pereira A., Rohlin M. (2011). Ambiguity in bone tissue characteristics as presented in studies on dental implant planning and placement: A systematic review. Clin. Oral Implant. Res..

[3] Holahan C., Wiens J., Weaver A., Assad D., Koka S. (2011). Relationship between systemic bone mineral density and local bone quality as effectors of dental implant survival. Clin. Implant. Dent. Relat. Res..

[4] Radi I., Ibrahim W., Iskandar S., AbdelNabi N. (2018). Prognosis of dental implants in patients with low bone density: A systematic review and meta-analysis. J. Prosthet. Dent..

[5] Staedt H., Rossa M., Lehmann K., Al-Nawas B., Kämmerer P., Heimes D. (2020). Potential risk factors for early and late dental implant failure: A retrospective clinical study on 9080 implants. Int. J. Implant. Dent..

[6] Grisa A., Veitz-Keenan A. (2018). Is osteoporosis a risk factor for implant survival or failure?. Evid. Based Dent..

[7] Nuţu E. (2018). Role of initial density distribution in simulations of bone remodeling around dental implants. Acta Bioeng. Biomech..

[8] Chrcanovic B., Albrektsson T., Wennerberg A. (2017). Bone quality and quantity and dental implant failure: A systematic review and meta-analysis. Int. J. Prosthodont..

[9] Lekholm U., Zarb G.A., Brånemark P.-I., Zarb G.A., Albrektsson T. (1985). Patient selection and preparation. Tissue Integrated Prostheses: Osseointegration in Clinical Dentistry.

[10] Rozé J., Babu S., Saffarzadeh A., Gayet-Delacroix M., Hoornaert A., Layrolle P. (2009). Correlating implant stability to bone structure. Clin. Oral Implant. Res..

[11] Heinemann F., Hasan I., Bourauel C., Biffar R., Mundt T. (2015). Bone stability around dental implants: Treatment related factors. Ann. Anat..

[12] Szmukler-Moncler S., Salama H., Reingewirtz Y., Dubruille J. (1998). Timing of loading and effect of micromotion on bone dental implant interface: Review of experimental literature. J. Biomed. Mater. Res..

[13] Alghamdi H. (2018). Methods to improve osseointegration of dental implants in low quality (Type-IV) bone: An overview. J. Funct. Biomater..

[14] Summers R. (1994). A new concept in maxillary implant surgery: The osteotome technique. Compendium.

[15] Hahn J. (1999). Clinical uses of osteotomes. J. Oral Implantol..

[16] Komarnyckyj O., London R. (1998). Osteotome single-stage dental implant placement with and without sinus elevation: A clinical report. Int. J. Oral Maxillofac. Implant..

[17] Nóbrega A., Norton A., Silva J., Silva J., Branco F., Anitua E. (2012). Osteotome versus conventional drilling technique for implant site preparation: A comparative study in the rabbit. Int. J. Periodontics Restor. Dent..

[18] García-Vives N., Andrés-García R., Rios-Santos V., Fernández-Palacín A., Bullón-Fernández P., Herrero-Climent M., Herrero-Climent F. (2009). In vitro evaluation of the type of implant bed preparation with osteotomes in bone type IV and its influence on the stability of two implant systems. Med. Oral Patol. Oral Cir. Bucal..

[19] Attanasio F., Antonelli A., Brancaccio Y., Averta F., Figliuzzi M.M., Fortunato L., Giudice A. (2020). Primary stability of three different osteotomy techniques in medullary bone: An in vitro study. Dent. J..

[20] Attanasio F., Bortolini S., Carbone D., Pacifici A. (2020). Flapless cone beam computed tomography-guided implant surgery with contextual transcrestal sinus lift augmentation using new bone compactor tools. Case Rep. Dent..

[21] Antonelli A., Bennardo F., Brancaccio Y., Barone S., Femiano F., Nucci N., Minervini G., Fortunato L., Attanasio F., Giudice A. (2020). Can bone compaction improve primary implant stability? An in vitro comparative study with osseodensification technique. Appl. Sci..

[22] Huwais S., Meyer E.G. (2017). A novel osseous densification approach in implant osteotomy preparation to increase biomechanical primary stability, bone mineral density, and bone-to-implant contact. Int. J. Oral Maxillofac. Implants.

[23] Lahens B., Neiva R., Tovar N., Alifarag A., Jimbo R., Bonfante E., Bowers M., Cuppini M., Freitas H., Witek L. (2016). Biomechanical and histologic basis of osseodensification drilling for endosteal implant placement in low density bone. An experimental study in sheep. J. Mech. Behav. Biomed. Mater..

[24] Alifarag A., Lopez C., Neiva R., Tovar N., Witek L., Coelho P. (2018). Atemporal osseointegration: Early biomechanical stability through osseodensification. J. Orthop. Res..

[25] Delgado-Ruiz R., Gold J., Somohano Marquez T., Romanos G. (2020). Under-drilling versus hybrid osseodensification technique: Differences in implant primary stability and bone density of the implant bed walls. Materials.

[26] Natali A., Carniel E., Pavan P. (2009). Dental implants press fit phenomena: Biomechanical analysis considering bone inelastic response. Dent. Mater..

[27] Frost H. (1989). The biology of fracture healing: An overview for clinicians. Part I. Clin. Orthop..

[28] Frost H. (1989). The biology of fracture healing: An overview for clinicians. Part II. Clin. Orthop..

[29] Shelley A., Glenny A., Goodwin M., Brunton P., Horner K. (2014). Conventional radiography and cross-sectional imaging when planning dental implants in the anterior edentulous mandible to support an overdenture: A systematic review. DentoMaxillofac. Radiol..

[30] Jacobs R. (2011). Dental cone beam CT and its justified use in oral health care. J. Belg. Radiol..

[31] Irie M., Rabelo G., Spin-Neto R., Dechichi P., Borges J., Soares P. (2018). Use of micro-computed tomography for bone evaluation in dentistry. Braz. Dent. J..

[32] Chang G., Boone S., Martel D., Rajapakse C., Hallyburton R., Valko M., Honig S., Regatte R. (2017). MRI assessment of bone structure and microarchitecture. J. Magn. Reson. Imaging.

[33] Singh S., Bray T., Hall-Craggs M. (2018). Quantifying bone structure, micro-architecture, and pathophysiology with MRI. Clin. Radiol..

[34] Del-Valle M., Lins E., Ana P. (2019). Assessment of simulated osteoporosis in alveolar bone using optical coherence tomography. J. Biophotonics.

[35] Huang D., Swanson E., Lin C., Schuman J., Stinson W., Chang W., Hee M., Flotte T., Gregory K., Puliafito C.A. (1991). Optical coherence tomography. Science.

[36] Bakhsh T., Sadr A., Shimada Y., Mandurah M., Hariri I., Alsayed E., Tagami J., Sumi Y. (2013). Concurrent evaluation of composite internal adaptation and bond strength in a class-I cavity. J. Dent..

[37] Ugryumova N., Matcher S., Attenburrow D. (2004). Measurement of bone mineral density via light scattering. Phys. Med. Biol..

